# A web resource for mining HLA associations with adverse drug reactions: HLA-ADR

**DOI:** 10.1093/database/baw069

**Published:** 2016-05-17

**Authors:** Gurpreet S. Ghattaoraya, Yenal Dundar, Faviel F. González-Galarza, Maria Helena Thomaz Maia, Eduardo José Melo Santos, Andréa Luciana Soares da Silva, Antony McCabe, Derek Middleton, Ana Alfirevic, Rumona Dickson, Andrew R. Jones

**Affiliations:** ^1^ Department of Molecular and Clinical Pharmacology, Institute of Translational Medicine; ^2^ Institute of Integrative Biology; ^3^ Liverpool Reviews and Implementation Group; ^4^ Hesketh Centre, Mersey Care NHS Trust, Southport, UK; ^5^ Center for Biomedical Research, Faculty of Medicine, Autonomous University of Coahuila, Torreon, Mexico; ^6^ Human and Medical Genetics, Institute of Biological Sciences, Federal University of Pará, Tucuruí, Brazil; ^7^ Transplant Immunology Laboratory, Royal Liverpool and Broadgreen University Hospital, Liverpool, UK; ^8^ Institute of Infection and Global Health, University of Liverpool, Liverpool, UK

## Abstract

Human leukocyte antigens (HLA) are an important family of genes involved in the immune system. Their primary function is to allow the host immune system to be able to distinguish between self and non-self peptides—e.g. derived from invading pathogens. However, these genes have also been implicated in immune-mediated adverse drug reactions (ADRs), presenting a problem to patients, clinicians and pharmaceutical companies. We have previously developed the Allele Frequency Net Database (AFND) that captures the allelic and haplotype frequencies for these HLA genes across many healthy populations from around the world. Here, we report the development and release of the HLA-ADR database that captures data from publications where HLA alleles and haplotypes have been associated with ADRs (e.g. Stevens–Johnson Syndrome/toxic epidermal necrolysis and drug-induced liver injury). HLA-ADR was created by using data obtained through systematic review of the literature and semi-automated literature mining. The database also draws on data already present in AFND allowing users to compare and analyze allele frequencies in both ADR patients and healthy populations. The HLA-ADR database provides clinicians and researchers with a centralized resource from which to investigate immune-mediated ADRs.

Database URL: http://www.allelefrequencies.net/hla-adr/.

## Introduction

Adverse drug reactions (ADRs) are a major problem faced by clinicians and pharmaceutical companies, demonstrated by the fact that approximately 6–7% of hospital admissions in the UK have been attributed to ADRs (1, 2). In addition, ∼3–5% of newly approved drugs in Europe and North America (approved between 2002–11 and 1990–09, respectively) were withdrawn for safety reasons, with hepatotoxicity (an ADR) being the second most commonly cited reason in both geographic regions after cardiovascular events (3, 4).

Historically, there have been two categories of ADRs: type A (‘on target’) and type B (‘off target’ or ‘idiosyncratic’) (5). Type A reactions were described as observed responses that augment the known pharmacological effects of the drug, are dose dependent and predictable. Typical causes for type A reactions include inter-individual variability in pharmacokinetics such as increased or impaired absorption, distribution, metabolism or excretion of the drug. Idiosyncratic or type B ADRs are described as rare, not seen in most people being treated within the standard therapeutic dose range, but can potentially cause severe morbidity and possibly death. They are thought to be immune mediated (6) and in many cases the human leukocyte antigen (HLA) genes have been associated with these reactions (7, 8). As a result of these associations, it has been argued that referring to type B reactions as ‘unpredictable’ and ‘not dose dependant’ is largely incorrect and the definition of ‘off-target’ ADRs should be updated to reflect this (9, 10). Because type B reactions only contribute to 20% of all ADRs (11), the manifestations of the adverse reactions may not be observed in patients until the late stages of drug development or after general release, after many patients have been exposed to the drug. As well as the risks to patients, the ADRs can lead to post-marketing product withdrawal and significant financial loss due to the huge investment in drug development and manufacturing demands.

Immune-mediated ADRs can be observed in specific ethnic groups and have been reported to be HLA allele associated (12). The HLA genes are the most polymorphic in the human genome, with 13 840 known alleles (in October 2015; 10 297 class I and 3543 class II alleles) (13). However, the HLA genetic component does not explain all the contributing aspects to ADRs and there are likely other components including environment (diet, smoking, alcohol consumption etc.), host risk factors such as BMI, genetics, co-morbidities and co-administered drugs, which may also contribute to the onset of ADRs (8, 14).

Due to the extremely high number of alleles for some of the HLA genes, a nomenclature of HLA alleles was developed (15). All HLA alleles are given an ‘HLA’ prefix followed by a hyphen and the name of the gene (e.g. ‘HLA-A’) this is then followed by an asterisk (*) and a series of grouped numbers or fields separated by colons (e.g. HLA-B*57:01). The first field of numbers refers to the allele group (antigen/serotype). Alleles belonging to the same antigenic group will share the same number. The second field of numbers defines the specific HLA protein (e.g. HLA-B*57:01 is a part of the same antigenic group as HLA-B*57:03, but the protein sequences for these alleles differ by two amino acid residues). Additional fields (e.g. HLA-A*01:01:01:01) refer to synonymous changes in the protein-coding DNA sequence, i.e. causing no change in the protein sequence (third group) or changes in intronic regions (fourth group). In the rest of the article, we typically refer to specific proteins (second field) for discussing HLA alleles.

In the field of pharmacogenomics, there are two main study approaches that are implemented when trying to determine the genetic components of HLA-induced ADRs. These are genome-wide association studies (16, 17) and case–control candidate gene studies (18, 19). Both approaches have helped to identify HLA alleles associated with increased risk of developing ADRs with some of the highest odds ratios in medical literature. One of the examples is an association (P$=3.13\times 10^{-27};OR=2504;\;95\%\;CI:\;126–49\;522$) between HLA-A*15:02 and the most severe form of carbamazepine-induced hypersensitivity termed Stevens–Johnson syndrome (SJS) (18). Another example includes an association between HLA-B*57:01 and abacavir-induced hypersensitivity where it has been demonstrated that the drug is able to bind non-covalently to the HLA-B*57:01 protein (12).

It has been reported that HLA-allele-associated ADRs are more prevalent in certain populations, e.g. there is greater frequency of reported cases of carbamazepine induced SJS associated with HLA-B*15:02 in Han Chinese populations as compared to Caucasians and ethnic Japanese or Koreans (18, 20)—an allele found at relatively high frequency in East and Southeast Asia. Conversely, the presence of the HLA-A*31:01 allele is an important predictor for carbamazepine hypersensitivity in individuals of European descent (21).

Given the rare occurrence of severe ADRs and huge racial variability in the frequency of HLA alleles, only a relatively small number of individuals are reported in most studies that investigate ADRs associated with HLAs, leading to statistical analysis with low power. Rarity of severe ADRs also leaves researchers with great challenges for predicting, diagnosing and treating ADRs during drug development and clinical trials. One of the methods of addressing the potential risk to the patient for drugs known to induce ADRs is to implement genetic screening before the administration of the drug to determine whether a patient carries a known high-risk allele for this treatment, and in such cases, provide the patient with an alternative treatment therapy. This approach has already been effective in reducing ADRs in HIV patients who were prescribed abacavir or epileptic patients treated with carbamazepine. Patients were screened for the risk alleles (HLA-B*57:01 or B*15:02, respectively) and, if positive, provided with alternative treatment, reducing the incidence of drug-hypersensitivity reactions (22, 23).

With the research into ADRs becoming more widespread, it has become ever more important for clinicians, researchers and pharmaceutical companies to keep up to date with the information that is available. To assist the HLA and pharmacogenetic community, we have developed a web-based database to synthesize the evidence and enable digital data access. To achieve this, we have performed a systematic review and collated data sets from the literature, designed and implemented a relational database and created webpages to allow users to access these data, called the HLA-ADR. The new database has been implemented within the wider Allele Frequency Net Database (AFND), which stores large collections of data on the allele and haplotype frequencies for healthy, worldwide populations (24), as well as modules for exploring immunogenetic disease associations (25). HLA-ADR provides a resource that not only facilitates meta-analyses but also enables users to further their investigations by using resources available with the main AFND website, e.g. on the incidence of particular HLA alleles/haplotypes in healthy worldwide populations.

## Materials and Methods

### Systematic review

In order to populate the database, we conducted a systematic review to identify studies that contained the relevant data. We developed a search protocol, using internationally accepted standards, which was followed during this systematic review. The details of the protocol are laid out below and include the search strategy, the inclusion/exclusion criteria, method of screening and data extraction.

#### Search strategy

Searches were performed using the Medline, HuGE Navigator (26) and EMBASE literature databases. Search strategies were developed for MEDLINE and EMBASE (full details on the search strategy are available in the Supplementary file). These searches aimed at capturing studies in relation to adverse drug reactions that were associated with HLA alleles. HuGE Navigator utilizes MeSH terms (Medical Subject Headings: a controlled vocabulary of keywords) within the searches and incorporates synonymous terms within the results, therefore, using the term ‘Drug Toxicity and HLA’ for interrogating this database was deemed sufficient to capture the appropriate studies for inclusion in this review. A supplementary search was also performed in Medline using specific drug names followed by the phrase ‘(hypersensitivity OR pharmacogenetics OR HLA)’. The drugs were selected based on those reported in the HuGE Navigator search and also from review articles (7, 8). The PubMed ID numbers (PMID) for all search results were collected into a list, and duplicate PMIDs were removed. Restrictions were applied to only search for publications written in English.

#### Eligibility criteria

Details of how eligible studies were determined are presented in Table 1. In short, only case–control studies that investigated ADRs in patients and provided statistical evidence of the association were included. In relation to HLA typing in these studies, only investigations where the HLA genes of the patient–control cohort were genotyped to protein level (‘second field resolution’ or above) were included. This was decided because it has been demonstrated, at least for abacavir, that alleles within the same antigenic group do not elicit the same ADR risk profile (12). As such, we feel the small number of studies with HLA typing to only the first field do not greatly contribute to understanding. However, studies were included if a mixture of high- and low-resolution typing had been performed. 

**Table 1.** Inclusion criteria for the literature review.

Population	Patients with a hypersensitivity reaction to drugs administered as part of standard treatment
Study design	Retrospective and prospective case-controlled studies, randomised controlled trials
Statistical evidence	Provided statistical evidence e.g. *P* values, OR (95% CI) to determine the strength of the association.
HLA typing	Investigations where patient and control HLA status was determined to the protein level (high resolution typing sometimes referred to as ‘four-digit resolution’). Studies where the HLA allele were genotype within the gene itself, i.e. not using proxy SNPs.
Study date	A formal systematic review was performed covering studies with the publication year between 2000 and the time of the search (April 2014) as described in the Materials and Methods section, followed by additional semi-automated searching covering April 2014 to Aug 2015 and also 1995 to 1999 (see additional studies).

In addition, we only included studies in which the HLA alleles were genotyped using any recognized method able to determine high-resolution allele calls with high confidence. We chose to exclude studies in which SNP-based proxy (tagging) methods were used, i.e. with SNPs outside the gene, elsewhere on the chromosome. (Proxy) SNP tagging methods have been shown to be unreliable across different populations because they rely on the SNP tag to be in perfect linkage disequilibrium with the allele of interest. For example, in the case of SNP rs2395029 as a marker for HLA-B*57:01 abacavir hypersensitivity, see (27) for NCBI dbSNP for these SNP designations, the tag SNP was reported to be in perfect linkage disequilibrium with the HLA-B*57:01 allele in a Mexican population (28); however, this was not seen across all populations (29, 30). In addition, HLA-DQA1*01:02, which has been associated (as part of a haplotype) with lumiracoxib hepatotoxicity in Caucasians, uses the SNPs rs9270986 and rs3129900 as proxy markers (31). However, when investigated in other populations, the use of these tags was shown to be limited (32). In fact, an investigation by de Bakker *et al.* (33) demonstrated that the same allele will require a different SNP tags in different populations, e.g. HLA-A*31:01, for carbamazepine hypersensitivity would require the tag SNPs: rs1061235 in Caucasians; rs3823318 and rs1061235 in Chinese and rs1061235 and rs1150739 in Japanese populations. Therefore, the clinical relevance of using this tag SNP is limited as multiple tags for each allele would be required to cover all populations and patient backgrounds will need to be taken into account in clinical settings. This would also limit the ability to perform meta-analyses on the data. As a result, a decision was taken to exclude studies where the patients HLA status was determined via tag SNPs.

More recently, technologies have been developed whereby HLA alleles can be imputed using multiple SNP positions—e.g. HIBAG (34). Earlier algorithms using similar method were limited as they required extremely large ethnic-based cohorts as training sets in order to make accurate predictions. Such training set data are not always available, as a result, alleles that are rare in these populations are difficult to call accurately (35). For this reason, we chose not to include studies that used the early methods of imputation in our systematic review. However, as newer algorithms, such as the above-mentioned HIBAG, are emerging, which are able to overcome these limitations, we may change our inclusion/exclusion criteria in future updates to the database to reflect these advances.

#### Literature screening

The studies passed through two stages of screening to determine eligibility. The first stage involved reviewing the titles and abstracts of the studies identified in the literature search to determine relevance. The second stage involved obtaining and examining the full paper to assess if they fulfilled the eligibility criteria. In circumstances where the full paper was unavailable (*n* = 22), the study was not included. The selected studies were also assessed by a second reviewer with discrepancies being resolved through discussion.

#### Data extraction

Data from the included studies were extracted to a spreadsheet document by three reviewers and independently checked by two reviewers. Examples of the types of data that were extracted are provided in Supplementary file 1. Additional notes that were considered relevant were also recorded—e.g. in some cases in the literature, the association is reported as less than the significance threshold (e.g. as ‘<0.05’), we report this as ‘0.05’ with a note stating this fact.

### Additional studies

Because the systematic review was conducted, 11 new studies have been published that meet the inclusion criteria of the systematic review and were identified using a follow-up semi-automated search. The data from these studies have been extracted and included in the HLA-ADR database. The semi-automated search involved re-conducting the supplementary Medline search as described in Materials and Methods section and applying date filters coving publications released between April 2014 and August 2015. The first-stage screening process was conducted using a computer script that analyzes the titles and abstracts of the studies for keywords (drug names, references to HLA alleles and references to ADRs) and highlighting these articles for the second-stage review process, which remained a manual process as described above. This search was also repeated for studies published between 1995 and 1999 where an additional five studies were identified, meaning a total of 16 studies were identified via this method. We intend to use the semi-automated process periodically to scan the literature for new data to insert, with follow-up (formal) systematic reviews every few years to ensure high data quality.

**Figure 1.**
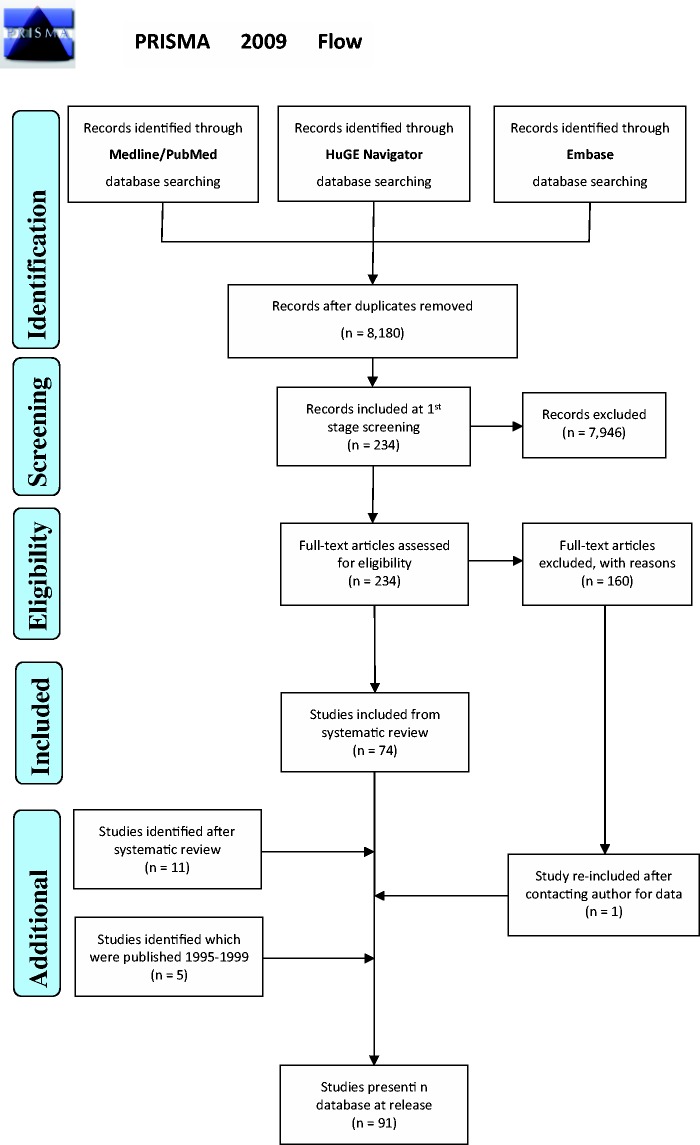
PRISMA 2009 Flow demonstrating the procedures followed for including data sets in HLA-ADR.

In addition, a study was initially rejected during the systematic review due to lack of data pertaining to the number of patients and controls carrying certain alleles (i.e. statistical evidence for the association). We contacted the authors requesting the required data; the requested information was supplied and therefore this study was included *post hoc* (Figure 1). 

### Web database

To enable users to query and retrieve the information, a web database was created. This involved inserting the extracted data into a relational database schema with interactive webpages built on top. The webpages were developed with Active Server Pages scripting environment that serves out HyperText Markup Language (HTML) and Cascading Style Sheets (CSS). This allows the data to be viewed using most common web browsers.

## Results

### Data

Seventy-four articles (plus one added *post hoc*) from the initial 7978 studies from this systematic review, and an additional 16 studies from semi-automated search, were eligible for inclusion (Figure 1) (36). From these 91 articles, all are case–control studies with 1350 ADR-allele comparisons (of which 221 were statistically significant associations) recorded into the database. The overall data cover 25 distinct drugs—carbamazepine being the most frequently reported (29 studies) and six drug groups were not separated when reported in the original study. The associations covered 16 different types of ADR phenotypes, which were tested against a total of 386 distinct HLA alleles (328 high-resolution alleles + 58 serotype/antigens) in the patient and control cohorts from these studies. The alleles represent 12 genes from the HLA family. A list of all the studies present in the database can be found on the website using the URL: http://www.allelefrequencies.net/hla-adr/adr_data_source.asp.

The demographic data from these studies showed that the investigations were predominantly performed on the Asian and European continents, representing 60% and 23% of the included studies, respectively. Because many of the investigations controlled for ethnicity by using patient cohorts comprised of just one ethnic group, it is not surprising that Asian and European cohorts made up the majority of the ethnic groups that are represented in the database.

### Website organization

Webpages created for accessing and submitting data to HLA-ADR were integrated into the AFND website and can be accessed either through the AFND main web-portal or directly with the URL: http://www.allelefrequencies.net/hla-adr. From this link, users have the option to view the HLA-ADR query page or the HLA-ADR reports page, and to submit studies to the database.

#### Query page

**Figure 2.**
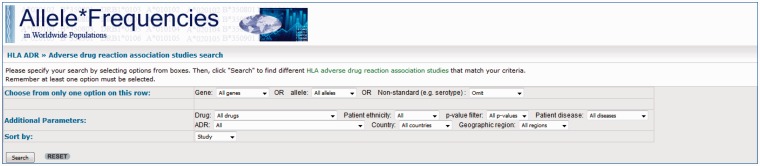
Screenshot of the HLA-ADR query page. Users can query the database via the use of filters. They may choose to see associations for genes/alleles, drugs, patient ethnicity, level of significance (*P* values) and the country/region where the study was conducted.

The query page allows users to retrieve data via the use of dropdown filters (Figure 2) where users may select associations with certain conditions. The dropdowns are divided into three sets; with the first set, users may choose the HLA gene, a specific allele or a non-standard allele (e.g. a serotype/antigen). The options within this set are mutually exclusive, meaning the user may only apply a filter from one of these although the user may use an option from this set in combination with filters from the other sets. The second set of options allow the user to specify additional parameters, specifically: a drug, patient ethnicity, strength of the association (*P* values), the country/region where the study was conducted or the condition for which the patients are being treated for (e.g. epilepsy). The filters from this set can be applied in combination with each other. The final set allows the users to choose which order they wish the data to be presented (e.g. group all the results from individual studies together, keep associated alleles together or by drug name). The use of dropdown options for this database allows for fine-tuning of the search allowing users to view associations matching their desired criteria. 

**Figure 3.**
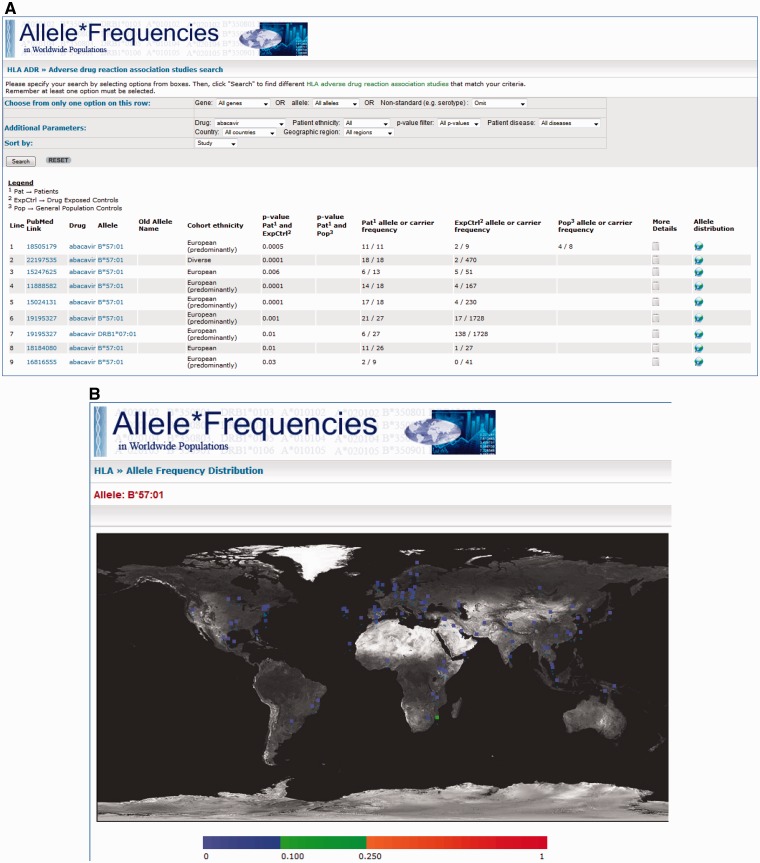
A, Screenshot of the results page where ‘abacavir’ was used as the search criteria. The results show the PubMed/MEDLINE link, the drug featured in the study, the tested allele for the associations, the patient/control cohort ethnicity, the statistical evidence, links to ‘More Details’ and AFND worldwide distributions for that allele. **B**, A screenshot generated by AFND showing worldwide distributions for the queried allele (e.g. HLA-B*57:01 for abacavir hypersensitivity). This page will be displayed by clicking on the allele distribution link seen in A.

Once the database is queried, the results are displayed in a table format with each row displaying information for each specific association. For simplicity of display, summary information about each record is provided: a link to the PubMed/Medline abstract for the original study, the drug, tested allele, the patient/control cohort ethnicity, the strength of the association and the number of patients and controls in the study cohort carrying the allele. A link is also provided (‘More Details’) whereby the complete data are shown for that specific association. A second link (‘Allele Distribution’) connects to the main AFND site showing the worldwide distribution of the allele on a map of the world (Figure 3). 

#### Reports page

**Figure 4.**
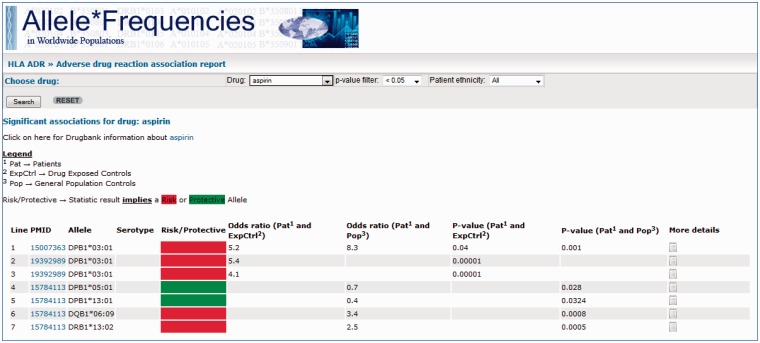
Screenshot of the AFND ADR reports page from a search for drug = aspirin, with the other options remaining at their default settings (*P* < 0.05 and no restrictions on patient ethnicity). A link is provided to the DrugBank page for the drug underneath the search bar. Much like the HLA-ADR query page, the summary table of results show the PubMed/MEDLINE link, the associated allele, the statistical data and a link to the ‘More details’ page where full details about the association can be found. In addition, a ‘risk/protective’ allele assignment is given (red or green box) based on the proportion of carriers of the allele in the patients group compared with the control group.

In addition to query page, an HLA ADR report page is also provided (Figure 4). Here, the webpage allows the user to select a particular drug and returns all database records pertaining to that drug, which are statistically significant (the user may select the significance threshold). An optional filter also enables filtering by the patient group. The returned entries are initially provided as a summary table—indicating alleles that have been reported, statistical significance values and whether the association implies that the allele is a risk or protective marker. 

In addition, the report provides relevant information embedded from other resources. First, for each allele, the worldwide distribution is displayed on a map. Second, search results (from AFND) for HLA haplotype frequencies (in which each allele can reside) are displayed. This feature is potentially useful for identifying if two different alleles reported to be associated with a given ADR are in linkage disequilibrium, and thus unlikely to be independent (different) causative risk factors. Multiple sequence alignments (MSA) powered by IMGT/HLA (13) are then provided for all of the reported alleles (grouped by genes). The MSA is performed using the alleles of the same gene from the summary table along with 10 common alleles for this gene to provide as comparison or controls. The purpose of this feature is to enable users to examine whether different alleles reported to be associated with a given ADR may share sequence similarities that are absent from other common (control) alleles. Finally, the report page embeds the IMGT/HLA allele report. This provides the user with information about the allele, such as publications reporting the allele along with the protein, nucleotide and genomic sequences for the allele.

#### Authors’ submission page

A webpage has been provided whereby authors may submit their own data. The page provides links to download a tutorial file and a spreadsheet document to complete. Once the data have been entered and saved onto the spreadsheet document, the user can upload the completed file using the authors’ submission page. We encourage researchers with data to include their own data, both published and non-published as well as data that show non-significant associations. We believe that this will improve the ability to perform meta-analyses using the database. This will also ensure that HLA-ADR stays up-to-date in this fast moving field.

## Discussion

It is evident that information relating to ADRs will continue to grow, and thus it is important that data sets and results are digitized to enable clinicians and researchers to access them from a centralized location. In response, we have performed a systematic review and developed a web-based database to provide flexible access. To ensure that the system can be maintained and kept up-to-date, we have added a data submission page enabling users to submit their own studies. In addition, as HLA-ADR is part of the wider AFND site (which has been running successfully for >10 years), the AFND team will now include ADR data in their regular cycles of curation.

We envisage that the database will predominantly be used in pharmacogenetics research, e.g. enabling scientists to perform meta-analyses in a straightforward manner using HLA-ADR as a single resource. Moreover, the links to external resources, such as to IMGT/HLA and the main AFND utilities will provide users with a starting point from which to conduct further informatics investigation. Users should be aware we display information as they appear in the original source study without further interpretation as to the reliability of a statistical association. We have only made changes, with notifications, when we considered it absolutely necessary (e.g. if the allele association in the study was reported with the old nomenclature system and can be unambiguously updated to the modern nomenclature). The risk/protective allele designation on the reports page is the only instance where we have placed an interpretation on the data. This was done to address the issue where certain associations are statistically significant, but are clinically immaterial (apparent protective alleles). Often, for the investigation of ADRs, these need to be treated differently to risk alleles, and as such, we felt it appropriate to highlight these. In a clinical setting, HLA-ADR provides clinicians access to information about worldwide/ethnicity-based distributions of alleles, and incidence of ADRs for a given drug that can aid in clinical decision making by enabling the potential risk to be assessed more effectively.

Although data from a relatively large number of studies have been entered to date, to ensure quality of data, we have applied very strict inclusion criteria. However, if we see sufficient demand from users, the database may be expanded to include studies previously not included, e.g. those that report alleles resolved to the antigen level (low resolution or to the first field). In addition, one current limitation to the database is that it does not include unpublished data—which could lead to publication bias. As a result, the database does not comprehensively cover all of the (potentially) available data. In order to address this, an author’s submission page for HLA-ADR database has been developed, and we encourage users to submit unpublished data sets, including those containing no significant associations, which are otherwise inaccessible to the wider research community. We will perform regular curation of literature ourselves to extract any studies not submitted directly from authors. We also welcome researchers and/or authors to directly contact us to include studies already published and those published after the release of HLA-ADR that we have missed during our data curation. Users can refer to out HLA-ADR database bibliography page to view the list of studies present in the database.

## Conclusion

With the amount of data in the pharmacogenetics field ever increasing, we believe that HLA-ADR database has the potential to assist ADR research and facilitate meta-analyses as well as aiding clinicians. We will continue to develop and expand upon the current query tools, based on user feedback, over the coming years. We have already provided a report page that we envisage will provide researchers with a starting point from which to conduct further research and eventually, aid in the understanding of the underlying mechanisms of ADRs. We are also in the process of developing additional tools for applications in clinical settings that will be released as this database is updated.

## Funding

University of Liverpool PhD studentship (G.G.). 

## Supplementary Material

Supplementary Data

Supplementary Data
