# Supplementary material for: A web resource for mining HLA associations with adverse drug reactions: HLA-ADR
Source: Database (Oxford). 2016 May 17;2016:baw069. doi: 10.1093/database/baw069 (PMC5647400; doi:10.1093/database/baw069)
Supplement: Supplementary Data [file supp_2016_baw069_index.html]

Supplementary Data 

# A web resource for mining HLA associations with adverse drug reactions: HLA-ADR

## Supplementary Data

files

- Supplementary Data - doc file
