# Supplementary material for: A web resource for mining HLA associations with adverse drug reactions: HLA-ADR
Source: Database (Oxford). 2016 May 17;2016:baw069. doi: 10.1093/database/baw069 (PMC5647400; doi:10.1093/database/baw069)
Supplement: Supplementary Data [file supp_baw069_Supplementary.doc]

# Supplementary

## Search strategy

### Medline

**Database: Ovid MEDLINE(R) In-Process & Other Non-Indexed Citations and Ovid MEDLINE(R) <1946 to Present>**

Search Strategy:

--------------------------------------------------------------------------------

1 (drug induced or drug-induced).ti,ab. (27831)

2 (adverse drug reaction$ or adverse reaction$).ti,ab. (30786)

3 exp Drug Hypersensitivity Syndrome/ or exp Drug Hypersensitivity/ (37955)

4 hypersensitivit$.ti,ab. (48656)

5 exp Pharmacogenetics/ (9386)

6 exp HLA Antigens/ (64053)

7 (human leukocyte antigen$ or HLA).ti,ab. (81597)

8 (pharmacogenetic$ or genetic$).ti,ab. (670906)

9 (Abacavir or Allopurinol or Aminopenicillin or Aspirin or Carbamazepine or Clozapine or Co-amoxiclav or Co-trimoxazole or d-Penicillamine or Diclofenac or Feprazone or Flucloxacillin or Gold Sodium or Thiomalate or Hydralazine or Lapatinib or Levamisole or Lumiracoxib or Methazolamide or Nevirapine or Oxcarbazepine or Oxicam or Phenytoin or Sulphamethoxazole or Ticlopidine or Trichloroethylene or Ximelagatran).af. (146055)

10 or/1-4 (133860)

11 or/5-8 (758141)

12 10 and 11 (5967)

13 9 and 12 (772)

14 (animals not (humans and animals)).sh. (3860455)

15 13 not 14 (759)

16 limit 15 to english language (696)

***************************

### EMBASE

**Database: Embase <1974 to 2014 June 20>**

Search Strategy:

--------------------------------------------------------------------------------

1 (drug induced or drug-induced).ti,ab. (35493)

2 (adverse drug reaction$ or adverse reaction$).ti,ab. (44767)

3 exp drug hypersensitivity/ (49292)

4 hypersensitivit$.ti,ab. (59577)

5 exp pharmacogenetics/ (21416)

6 exp HLA antigen/ (80485)

7 (human leukocyte antigen$ or HLA).ti,ab. (103869)

8 (pharmacogenetic$ or genetic$).ti,ab. (781699)

9 (Abacavir or Allopurinol or Aminopenicillin or Aspirin or Carbamazepine or Clozapine or Co-amoxiclav or Co-trimoxazole or d-Penicillamine or Diclofenac or Feprazone or Flucloxacillin or Gold Sodium or Thiomalate or Hydralazine or Lapatinib or Levamisole or Lumiracoxib or Methazolamide or Nevirapine or Oxcarbazepine or Oxicam or Phenytoin or Sulphamethoxazole or Ticlopidine or Trichloroethylene or Ximelagatran).af. (336136)

10 or/1-4 (174284)

11 or/5-8 (902379)

12 10 and 11 (8682)

13 9 and 12 (1899)

14 limit 13 to (human and english language) (1596)

***************************

## Details of clinical data extraction

Data extraction will include, but may not be limited to:

**Study details**

- Bibliographic information/bibtex reference
- PubMed ID – to provide link back to original manuscript
- Corresponding authors and email
- Disease (that patients were initially treated for)
- Drug administered

**Study cohort demographic**

- Country
- City & province/state
- Group sizes (patients, controls)
- Ethnicity

**Association data**

- Allele
- Induced phenotype(s)
- Frequency of allele within each sample group
- Odds ratio (with confidence intervals)
- P-values
- Statistical method used for analysis (and any applied corrections)
